# Mutation and aging: news from the pool

**DOI:** 10.18632/aging.204779

**Published:** 2023-05-30

**Authors:** Francesca Marcon, Margherita Bignami, Peter Karran

**Affiliations:** 1 Department of Environment and Health, Istituto Superiore di Sanità, Rome, Italy; 2 Istituto Superiore di Sanità, Rome, Italy; 3 Francis Crick Institute, London, UK

**Keywords:** deoxynucleotide triphosphate pool, hMTH1, oxidative stress, lifespan, protein misfolding

The universality and inevitability of the aging process suggests that the underlying mechanisms are likely to be similar across animal species and that data from animal models can therefore potentially inform human aging. Recent experiments using an engineered mouse strain indicate a plausible role for the early acquisition of oxygen-related mutations in the aging process. Altered mitochondrial function and increased steady-state levels of reactive oxygen species (ROS) feature in several models of cellular senescence and aging. In general, cellular macromolecules (principally the genome and the proteome) are regarded as the targets for damage by ROS. The nucleotide pool that provides DNA replication precursors is also a significant ROS target and oxidation damage to deoxynucleoside triphosphates is now implicated in influencing longevity. The major product of oxidation in the nucleotide pool – 8-oxo-7,8-dihydrodeoxyguanosine triphosphate (8-oxodGTP) – is mutagenic. Human cells encode a nucleotide hydrolase (hMTH1), the homolog of the *E. coli* MutT protein, that degrades 8-oxodGTP to prevent the incorporation of potentially miscoding 8-oxodG during DNA replication [1]. In *E. coli*, MutT counteracts spontaneous mutagenesis and *mutT* mutants have one of the highest known spontaneous mutation rates. Consistent with a similarly powerful antimutagenic role, enhanced nucleotide pool editing in human or mouse cells overexpressing hMTH1 significantly reduces the extremely high spontaneous mutation rates that result from a defect in DNA mismatch repair [2]. hMTH1 also has an unexpected role in counteracting cellular senescence and aging. It mitigates oncogene-induced senescence in cultured human fibroblasts [3] and its overexpression in mouse embryo fibroblasts delays the onset of senescence [4]. In addition, *in vivo* overexpression of hMTH1 in transgenic C57BL/6J mice (hMTH1-Tg mice) protects them against age-related increases in micronucleus frequency [5], a marker of aging in humans [6]. Strikingly, hMTH1-Tg mice have a significantly extended lifespan and importantly, older hMTH1-Tg mice retain many of the behavioural traits of young mice [4].

hMTH1 overexpression in young hMTH1-Tg mice has a surprising impact on their general metabolism - including mitochondrial metabolism and the TCA cycle [5]. At two months of age, hMTH1-Tg mice have a more active liver metabolism than their wild-type (WT) counterparts. This suggests that when maintained on a normal laboratory diet, young WT mice suffer stress related to oxidized DNA precursors. This possibility is confirmed by the finding that a brief (5 week) switch to a high fat diet (HFD, an acknowledged source of oxidative stress) at two months old further amplifies the metabolic differences between hMTH1-Tg and WT mice. In the WT mice, HFD-induced changes in metabolic rates are accompanied by mitochondrial dysfunction and excessive weight gain. Additional HFD-induced changes include acknowledged indicators of systemic oxidative stress including increased serum protein and lipid oxidation, and increased ROS-related DNA damage. hMTH1 overexpression protects against both the standard diet and HFD-induced oxidative stress in young mice.

The protective effect of hMTH1 overexpression is confined to young mice and the behaviour of adult (7-month-old) animals is strikingly different. By this age WT mice maintained on a normal laboratory diet appear free of stress related to nucleotide precursor oxidation and hMTH1 overexpression in the hMTH1-Tg mice has no detectable effect on their liver metabolome. Furthermore, a 5-week HFD challenge to these adult mice elicits little in the way of metabolic changes or other oxidative stress markers. The responses of adult WT and hMTH1-Tg mice are closely similar and the protective effect of hMTH1 overexpression is redundant in these adult mice.

The canonical nucleotide hydrolase activity of hMTH1 is antimutagenic. Since the effects of its overexpression appear to be largely confined to young mice, it seems likely that this is related to the developmental pattern of these mice. The birth weights, subsequent growth rates and adult bodyweights of WT and hMTH1-Tg mice are indistinguishable. In both genotypes, growth is extremely rapid between conception and around 10 weeks after birth during which time the animals attain more than 80% of their final body mass [5]. In WT mice these early weeks are associated with an oxidative stress against which hMTH1 protects. This period of explosive growth and maximum DNA replication is a time when the purity of the nucleotide pool is paramount. It is likely that the mild oxidative stress experienced by young WT mice on a standard laboratory diet is associated with the accumulation of oxidation-related mutations. The hMTH1-Tg mice will accumulate fewer mutations of this kind. Might this explain their increased longevity?

The involvement of mutagenesis in aging is not straightforward. Aging affects everyone. By analogy, although only approximately one in three people will be diagnosed with cancer – a mutation-driven pathology - all three individuals will inevitably succumb to aging. Clearly the cancer paradigm of selection of key ‘driver’ mutations is unlikely to apply to aging. An alternative possibility is that all mutated genes are potential contributors to aging. They might do this via detrimental effects on the proteome that worsen with time. The altered primary sequence of a protein expressed from a mutated gene increases its risk of misfolding and misfolded proteins are highly susceptible to oxidation and aggregation. The powerful impairment of cellular systems by misfolded and oxidized proteins has been extensively documented in *in vitro* models [7]. Protein oxidation (carbonylation) and aggregation increase with age in mouse tissues [8]. On this model, aging is partly driven by a time-dependent accumulation of toxic protein aggregates that compromise cellular functions. As an example, the age-dependent increase in micronucleated cells in WT mice would then reflect a time-dependent degradation of DNA double strand break repair system(s) and/or factors affecting the accuracy of chromosome segregation. At least in these laboratory mice, toxic aggregates are formed from misfolded, oxidized proteins encoded by genes bearing oxygen damage-related mutations acquired during the first few weeks of development. Their aging is partly an inescapable legacy of events that occur in a brief period prior to adulthood.

The enhanced longevity of hMTH1-Tg mice is consistent with the prevention of early-life ROS-related mutations via nucleotide pool cleansing. It remains to be experimentally determined whether a reduced mutational burden extends their healthy lifespan by slowing the time-dependent accumulation of toxic misfolded and oxidized protein aggregates.
